# The Use of a Replicating Virus Vector For *in Planta* Generation of Tobacco Mosaic Virus Nanorods Suitable For Metallization

**DOI:** 10.3389/fbioe.2022.877361

**Published:** 2022-04-26

**Authors:** Keith Saunders, Eva C. Thuenemann, Sachin N. Shah, Hadrien Peyret, Ruth Kristianingsih, Sergio G. Lopez, Jake Richardson, George P. Lomonossoff

**Affiliations:** ^1^ Biochemistry and Metabolism, John Innes Centre, Norwich Research Park, Norwich, United Kingdom; ^2^ Computational and Systems Biology, John Innes Centre, Norwich Research Park, Norwich, United Kingdom; ^3^ Cell and Developmental Biology, John Innes Centre, Norwich Research Park, Norwich, United Kingdom

**Keywords:** TMV, viral coat protein, nanorod UI interface, replicating RNA, pEff, metallization, nanoparticles, Python script

## Abstract

The production of designer-length tobacco mosaic virus (TMV) nanorods in plants has been problematic in terms of yields, particularly when modified coat protein subunits are incorporated. To address this, we have investigated the use of a replicating potato virus X-based vector (pEff) to express defined length nanorods containing either wild-type or modified versions of the TMV coat protein. This system has previously been shown to be an efficient method for producing virus-like particles of filamentous plant viruses. The length of the resulting TMV nanorods can be controlled by varying the length of the encapsidated RNA. Nanorod lengths were analyzed with a custom-written Python computer script coupled with the Nanorod UI user interface script, thereby generating histograms of particle length. In addition, nanorod variants were produced by incorporating coat protein subunits presenting metal-binding peptides at their C-termini. We demonstrate the utility of this approach by generating nanorods that bind colloidal gold nanoparticles.

## Introduction

The tobacco mosaic virus (TMV) particle, a rigid nucleoprotein cylinder 300 nm in length with external and internal diameters of 18 and 4 nm, respectively, has been extensively studied for applications in nanobiotechnology (Lee et al., 2021). Chemical modification of the surface-exposed amino acids of wild-type TMV coat protein (CP) or genetically engineered changes to the CP can be used to produce particles with the modified properties (Shenton et al., 1999; Knez, et al., 2004; Bromley et al., 2008; Shah et al., 2016). Such a genetic modification enables gold cluster deposition on the exterior surface of particles (Lee et al., 2005). However, chemical modification to wild-type CP is limited to those naturally exposed amino acids that reside on the cylinder outer surface while incorporation of modified subunits often leads to diminished viral yields. The external and internal capsids of TMV particles have also been employed as a template for the formation of metallic nanotubes and nanowires *in vitro* (Shah, et al., 2014; Knez, et al., 2003) and electrostatic interactions have been used to bind 6 nm colloidal gold nanoparticles to wild-type TMV particles under acidic conditions (Khan et al., 2013). Bio-inorganic complex nanocomposites and specific metallic nanostructures have also been reported for applications in data storage, conductive and electrochemical processes in nanodevices, and in nanoelectronics (Gerasoloulos et al., 2012). Metal binding, coating and deposition can be achieved using metal salt precursors and bio-engineered CPs as previously reported (Shah et al., 2009; Steinmetz et al., 2009; Shah et al., 2014; Saunders and Lomonossoff, 2017).

One hindrance to the development of TMV-based technologies is the largely invariant length of the TMV particle produced via infection. This is determined by the length of the genomic RNA (6,395 nucleotides for the U1 or *Vulgare* strain of the virus) as each coat protein subunit interacts with three nucleotides. Thus, it is potentially possible to control the length of TMV rods by varying the length of the RNA to be encapsidated. However, the production of particles with lengths dramatically different from wild-type (300 nm) would require the use of RNA molecules significantly different in length from wild-type RNA, precluding the use of infection for their production. Thus, several alternative methods for producing defined length nanorods have been investigated, including *in vitro* assembly, expression of the coat protein and RNA containing the origin of assembly sequence (OAS) in heterologous hosts and the use of transient expression in plants (Saunders and Lomonossoff, 2017; Lomonossoff and Wege, 2018). However, these methods have limitations in terms of either the homogeneity or the amount of material that can be produced. Recently a replicating vector (pEff) based on potato virus X (PVX; Mardanova et al., 2017) has been shown to be highly effective at producing virus-like particles (VLPs) of several helical plant viruses, including TMV (Thuenemann et al., 2021). The VLPs contained RNA derived from the vector, the length of which can potentially be used to control the length of the nanorods. The yield of TMV nanorods was estimated to be at least a thousand-fold greater than that previously obtained with a non-replicating expression system (Saunders and Lomonossoff, 2017). We now report the development of this expression system to generate defined length nanorods containing either wild-type or modified coat protein subunits in sufficient quantities for bionanotechnological applications. These were characterized using a number of approaches, including a bespoke Python computer script coupled to the Nanorod UI interface script (https://ruthkr.shinyapps.io/nanorod) to analyze their length distribution.

## Materials and Methods

### Plasmids

**FIGURE 1:**
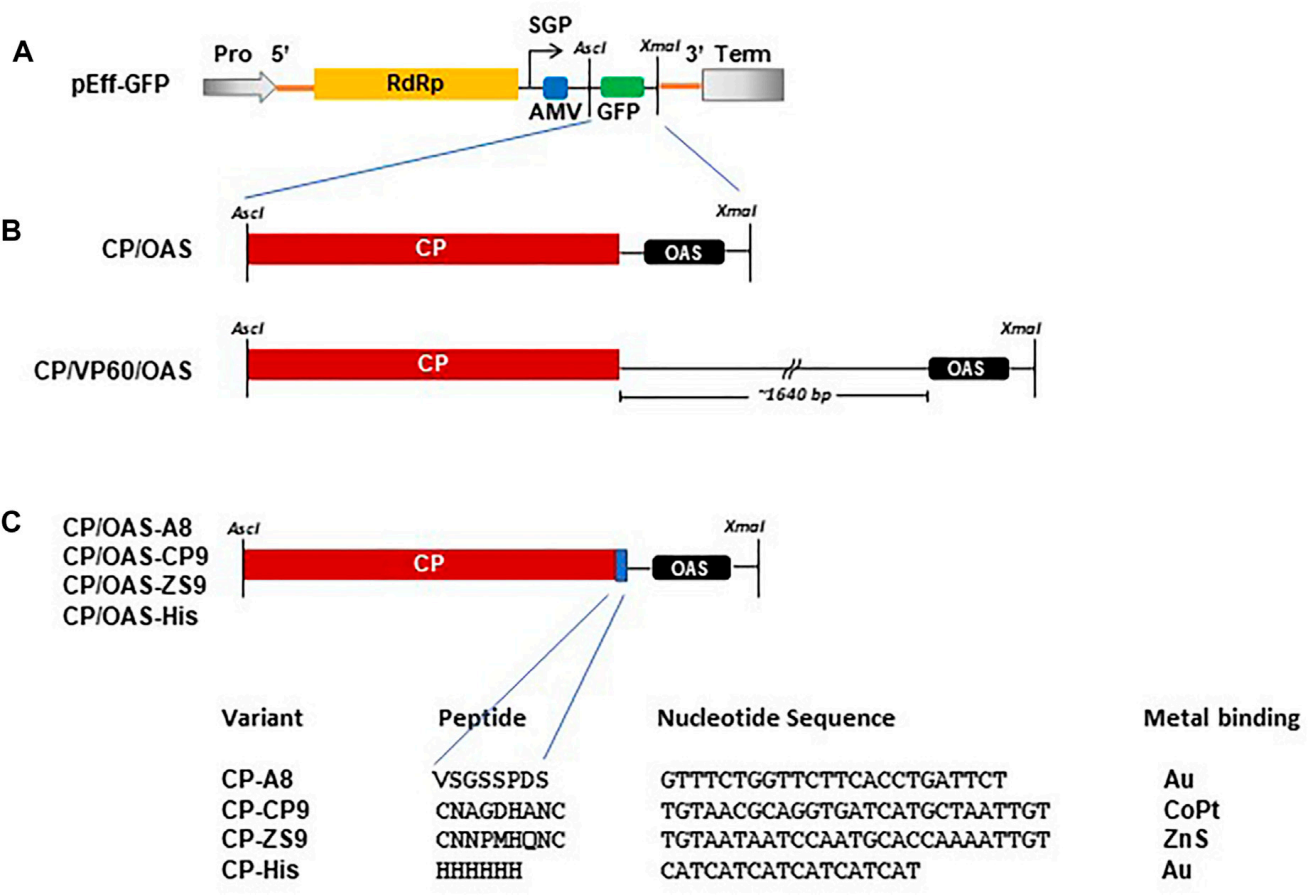
Schematic of constructs used in this study. **(A)** Diagrammatic illustration of the pEff vector showing the *Asc*I and *Xma*I cloning restriction sites located in the subgenomic region. Pro, 35S promotor. Term, *Nos* transcription terminator. SGP, subgenomic promotor. Yellow box RdRp, blue box AMV leader sequence and green box, GFP. **(B)** Arrangement of TMV CP with respect to the TMV OAS in CP/OAS and CP/VP60/OAS cloned into the subgenomic region of the pEff vector in place of GFP. **(C)** Variant TMV CPs indicating the additional amino acid residues located at the TMV CP C-terminus, the additional nucleotide sequence and the target bound metal.

The construction of pEff-TMV-CP/OAS, incorporating the sequence of the coat protein and the OAS of the *Vulgare* strain of TMV (Goelet et al., 1982; Saunders and Lomonossoff, 2017; GenBank accession number V01408.1) was described previously (Thuenemann et al., 2021; Figure 1). pEff-CP/VP60/OAS was produced by amplifying the TMV-specific region of pEAQ-HT-CP/VP60/OAS (Saunders and Lomonossoff, 2017) using end-tailoring primers to introduce an upstream AscI site and a downstream XmaI followed by cloning into pEff-GFP (GenBank accession number KY439904, Figures 1A,B; Mardanova et al., 2017; Thuenemann et al., 2021) in place of the sequence encoding GFP. DNA sequences (Thermo Fisher Scientific GENE-ART GmBH, Regensburg, Germany) encoding TMV coat protein with C-terminal additions (Figure 1C) and the OAS, corresponding to nucleotides 5,420–5,546 of TMV RNA (Goelet et al., 1982), were initially cloned into pEAQ-HT (GenBank accession number GQ497234.1, Sainsbury et al., 2009) via its AgeI and XhoI sites. These were subsequently amplified as described above and cloned into pEff-GFP via its AscI and XmaI restriction sites resulting in the replacement of the GFP sequence. All plasmids were electroporated into *Agrobacterium tumefaciens* strain LBA4404 as described by Sainsbury et al. (2009).

### Transient Expression

Samples were syringe-infiltrated into *Nicotiana benthamiana* leaves 21–28 days after pricking out. These were harvested 6–8 days post infiltration as previously described by Saunders and Lomonossoff (2017). Infiltrated leaves were homogenized in three volumes of extraction buffer (100 mM sodium phosphate, pH 7.0). After squeezing through Miracloth (Calbiochem), the crude extracts were clarified by centrifugation at 13,000 x *g* for 20 min at 4°C yielding the clarified fraction. This supernatant was incubated with one quarter volume of CHCl_3_, mixed and an additional centrifugation step of 13,000 x *g* for 20 min at 4°C was performed to separate the phases. One quarter volume of a 20% (w/v) PEG 6000, 1 M NaCl solution was added to the supernatant (CHCl_3_ fraction) and the mixture was stirred overnight in a cold room and a further sample was taken (PEG pre-clarification). Nanorods were sedimented at 13,000 x *g* for 20 min at 4°C and the pellet was re-suspended in 10 mM sodium phosphate pH 7.0 to yield the PEG post-clarification fraction. The preparations were further clarified by centrifugation at 27,000 x *g* for 20 min at 4°C. Nanorods in the resulting supernatant were recovered by centrifugation at 118,700 x *g* for 3 h at 4°C, suspended in 10 mM sodium phosphate pH 7.0 and applied to sucrose gradients. Nanorod concentrations were determined by absorbance at 260 nm using the value that a 1 mg/ml TMV solution has an absorbance of 3.0 (Brakke, 1967). RNA preparation for denaturing agarose gel electrophoresis and the determination of nanorod yield by protein assay (Pierce BCA protein assay, Thermo Fisher Scientific) were performed as previously described by Kruse et al. (2019) and Thuenemann et al. (2021).

### Sucrose Gradient Analysis

Nanorod preparations were subjected to centrifugation through a 15–30% (w/v) linear sucrose gradient buffered with 10 mM sodium phosphate, pH 7.0 prepared with the use of a BioComp Gradient Master Model 108 (BioComp Instruments Inc., Frederiction, New Brunswick, Canada) according to accompanying instructions. Ultracentrifugation was at 35,000 rpm, for 2 h at 4°C in a Sorvall TH641 rotor. After, gradients were fractionated with the use of a BioComp Model 152 piston gradient fractionator and the resulting fractions recorded with a BioComp Triax flow cell set at 280 nm absorption. Selected fractions, corresponding to peaks, volume 450 μL, were spun-washed at 1,000 x g in Millipore Amicon Ultra-4 100k MWCO spin filter units with several changes of 10 mM sodium phosphate buffer pH 7.0 and the volume reduced to 50 μL. Finally, fractions were resolved by NuPAGE-MOPS electrophoresis and imaged by transmission electron microscopy. As a control, a preparation of TMV (labelled 26 mg/ml and dated 10 September 1981, prepared at the MRC Laboratory of Molecular Biology, Cambridge, United Kingdom) that had been stored under cold room conditions, was subjected to ultracentrifugation as described above.

### Transmission Electron Microscopy

Nanorod preparations were diluted in water to approximately 0.1 mg/ml and 3.5 μL samples were placed on a Formvar carbon-filmed, 400 mesh copper grid (EM Resolutions, Sheffield, United Kingdom) which had been glow-discharged for 20 s at 10 mA in an Ace 200 (Leica Microsystems (United Kingdom) Ltd., Milton Keynes, United Kingdom). After 60 s, excess sample was wicked away using Whatman No. 1 filter paper and grids were negatively stained using 2% (w/v) uranyl acetate in H_2_O. Grids were imaged using a Talos F200C transmission electron microscope (Thermo Fisher Scientific, Eindhoven, Netherlands) operated at 200 kV, equipped with a 4 k OneView CMOS detector (Gatan United Kingdom, Abingdon, Oxfordshire, United Kingdom). Automated data acquisition was setup using EPU v 2.7.0.5806 (Thermo Fisher Scientific, Eindhoven, Netherlands), each image had a 1 s exposure with a sample dose of approximately 40 e^-^/A^2^, defocus of –1 μm, nominal magnification of ×45,000 and a calculated pixel size of 3.482 Å.

### Computational Methods for the Measurement of Nanorods

The nanorods were identified and measured using a custom-made image-processing pipeline written in Python. Python is a high-level programming language distributed under the GNU public license [Anaconda Software Distribution. Computer software. Vers. 3.8.10. Anaconda. 2016. Web. <https://anaconda.com>]. The Python libraries used in the pipeline are NumPy (Oliphant, 2006), Pandas (McKinney et al., 2010), Matplotlib (Hunter, 2007), Scikit-image (Van der Walt, et al., 2014), SciPy (Jones et al., 2001) and mrcfile (Burnley et al., 2017). Each image was transformed into a binary image using adaptive thresholding and a block size of 301 pixels. The binary image was then subjected to an erosion step and objects comprising less than 2000 pixels were removed. Subsequently, the image was dilated five times and holes with a size of less than 500 pixels were removed from the objects. The image was then watershed to separate close-lying objects. All objects with an area of less than 500 nm^2^ and a minor axis length of more than 40 nm were eliminated. Finally, the Feret maximum diameter of the objects, which is likely to be the distance between the two most distant corners of the nanorod, was used to calculate the length of each nanorod using Pythagoras’ theorem and the known width of the nanorods, 18 nm.

### Implementation of Measurement of Nanorods Scripts Into a User Interface

To make the Python measurement script more accessible, a user interface Nanorod UI was developed using the R programming language (version 4.1.0) (R Core Team, 2021). The Nanorod UI interface was built and deployed using the R Shiny package (Chang et al., 2021). The package Reticulate (Ushey et al., 2021) was used to allow interoperability or integration between the Python script and the R implementation. The results of the nanorod measurement then were read and analysed using the packages dplyr (Wickham et al., 2021), tidyr (Wickham, 2021), and readxl (Wickham and Bryan, 2019). To visualise the measurement results, other packages such as colourpicker (Attali, 2020), ggplot2 (Wickham, 2016), and DT (Xie et al., 2021) were used. After nanorod measurement, the users can manually discard rogue nanorod measurements and engage with the analysis process. Users can directly obtain the descriptive analysis and plot the histogram or frequency distribution of the data. The Nanorod UI interface is available at https://ruthkr.shinyapps.io/nanorod. The R Shiny code, as well as the Python script and its integration to R Shiny can be found in the GitHub repository https://github.com/ruthkr/nanorod.

### Binding of Colloidal Gold Nanoparticle to pEff-CP/OAS-A8 Nanorods

Unfractionated CP/OAS-A8 and CP/OAS nanorods, and TMV were dialysed against Milli-Q water for 4 h. A sample of 100 μL of a 10 μg/ml nanorods suspension was incubated for 3 min in an ultra-sonication bath running at 50–60 Hz. 15 μL of commercial gold particles, diameter 5 nm, OD1 (Sigma-Aldrich) stabilised in citrate buffer were added and the nanorods and gold nanoparticles were mixed before further ultra-sonication for an additional 3 min. The nanorod-gold particle suspension was transferred to a magnetic stirring base, set at 200 rpm, and incubated with constant stirring for a further 4 h followed by dialysis against two changes of Milli-Q water for a total of 8 h in a 10K MWCO Slide-A-Lyzer mini dialysis unit. All binding and dialysis reactions were carried out at 25°C. 4 μL aliquots of the dialysed reaction were deposited onto glow-discharged, Formvar copper grids coated with carbon film (C400 Cu100, EM Resolutions) and were imaged, either stained with uranyl acetate or unstained, by transmission electron microscopy.

## Results

### Tobacco Mosaic Virus Nanorods Are Synthesized Efficiently Using the pEff Vector

Our previous research has shown that synthesis of nanorods from pEff-CP/OAS results in the encapsidation of two distinct RNAs of approximately 1 kb and 5–6 kb (Thuenemann et al., 2021). The shorter RNA species represents the subgenomic RNA from which the coat protein is translated, while the larger represents the full-length PVX replicon that encodes the PVX RNA-dependent RNA polymerase (RdRp). Because each RNA possesses the TMV OAS sequence element located downstream of the region encoding the CP, both RNAs will be encapsidated by TMV CP leading to the generation of nanorods of expected lengths 49 and 257 nm. Similarly, nanorods generated from pEff-CP/VP60/OAS, in which 1,640 nucleotides derived from the CPMV VP60 gene (Saunders et al., 2009) (Figure 1B) were inserted between the TMV CP and the OAS sequences, should generate nanorods of the expected lengths of 129 and 339 nm. We will refer to nanorods as either CP/OAS or CP/VP60/OAS, dependent upon on their respective pEff constructs.

**FIGURE 2:**
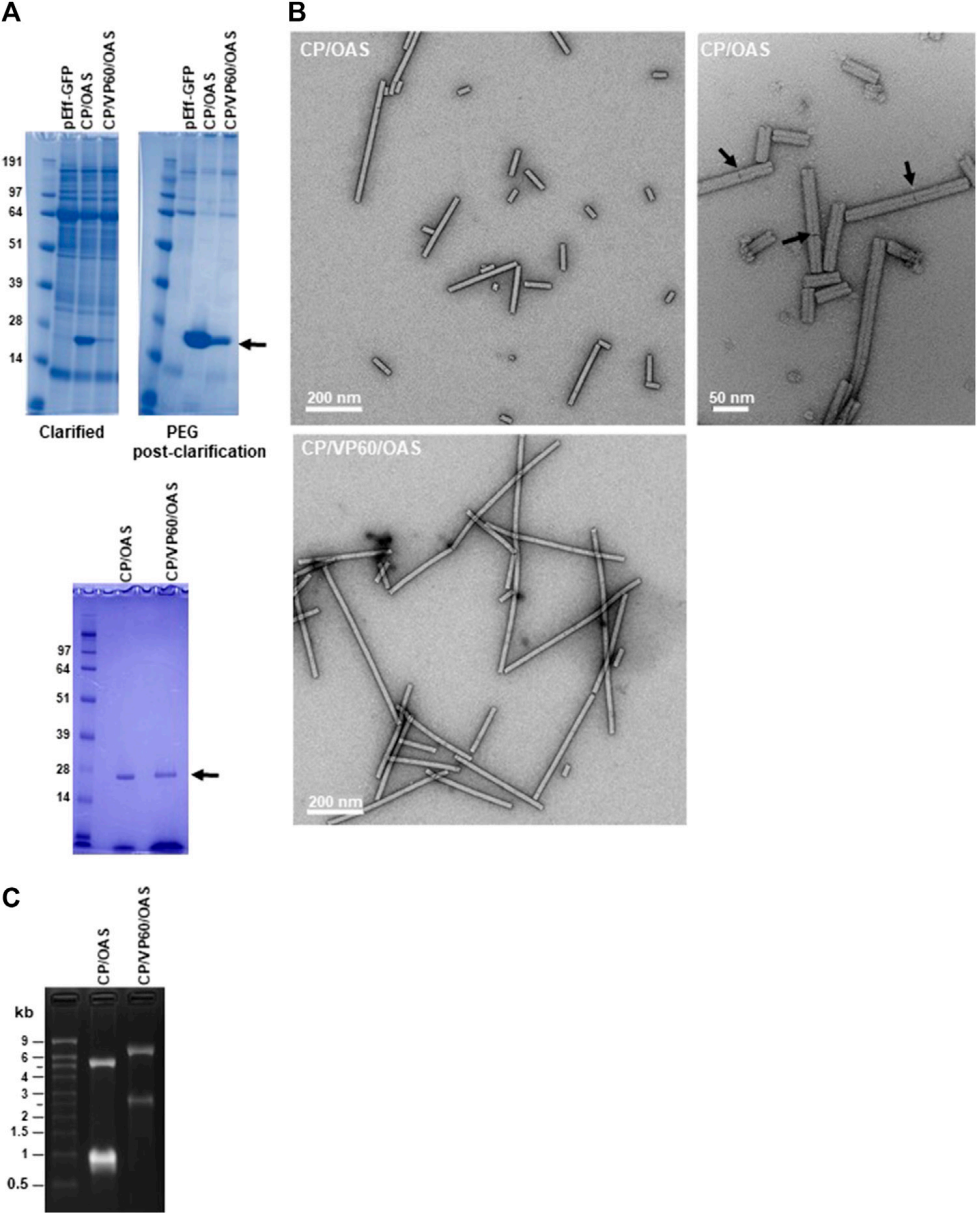
TMV nanorods produced using the pEff vector accumulate in infiltrated leaves. **(A)** Upper panels clarified extract (left) and PEG post-clarification (right) samples of CP/OAS and CP/VP60/OAS separated by NuPAGE-MOPS electrophoresis and stained with Instant Blue protein stain. Lower panel, samples fractionated prior to sucrose-density ultracentrifugation. Protein bands corresponding to TMV CP are indicated by an arrow. Further details can be found in Supplementary Figure S2. **(B)** Transmission electron microscopy images of nanorods of CP/OAS upper panels and of CP/VP60/OAS, lower panel. Arrows indicate differential uranyl acetate staining revealing end-to-end nanorod orientation. Scale bar 50, right panel or 200 nm, left panels as shown. **(C)** Ethidium bromide-stained agarose gel of RNA extracted from CP/OAS and CP/VP60/OAS nanorods. M, RNA size ladder.

To verify that infiltration with the pEff-based constructs gives rise to the expected lengths of nanorods, material extracted from infiltrated *N. benthamiana* leaves was analysed at different stages of the purification process. The presence of TMV CP in both clarified extracts and the PEG post clarification fractions was determined after denaturation by electrophoresis through NuPAGE-MOPS gels followed by Instant Blue staining. This revealed the presence of a single band of 18 kDa (arrowed), corresponding to the size of TMV CP in samples from both constructs (Figure 2A, upper panels). The amount of TMV CP was clearly greater in the CP/OAS preparation than in the CP/VP60/OAS preparation, a finding similar to that of a previous study when these corresponding gene constructs were expressed using the non-replicating pEAQ-*HT* vector system (Saunders and Lomonossoff, 2017). The yield of purified nanorods was assessed by UV/vis spectrometry. Typically, 25 g of infiltrated leaf material yielded 0.408 mg of nanorods in the case of CP/OAS compared to 0.063 mg in the case of CP/VP60/OAS nanorods. From this analysis, expression of CP/OAS nanorods via pEff expression was approximately 2,500 times greater than that achieved with the pEAQ-*HT* system (Saunders and Lomonossoff, 2017). The purity of the final nanorod preparations was confirmed by analysis of their protein content (Figure 2A, lower panel).

Transmission electron microscopy of the purified CP/OAS and CP/VP60/OAS nanorods showed they had a similar morphology to natural TMV (Symington et al., 1962), but exhibiting variations in length (Figure 2B). Nanorod length heterogenicity was seen particularly in the CP/OAS sample. Examination at high magnification suggests that the cause of this unexpected heterogeneity is end-to-end aggregation of the nanorods, since discontinuities could be seen in the longer rods (see arrowed examples of Figure 2B right panel). To confirm that the CP/OAS and the CP/VP60/OAS nanorods contained the anticipated lengths of RNA, RNA extracted from each preparation was analysed by electrophoresis on denaturing agarose gels followed by ethidium bromide staining. In each case, two RNA species of the expected lengths were resolved (Figure 2C). As previously reported (Thuenemann et al., 2021), CP/OAS preparations contained RNAs of approximately 5.5 and 1.0 kb while those of CP/VP60/OAS infiltrations appeared to each be about 1.5 kb longer, consistent with the insertion of 1,640 nucleotides downstream of the CP-encoding region in the latter case (Figures 1A,B). The fact that two discrete classes of RNAs can be resolved for both gene constructs supports the hypothesis that only these RNAs determine the length of the particles and that the heterogeneity in the length of nanorods observed by electron microscopy is not due to the encapsidation of additional, heterogeneous RNA molecules. End-to-end aggregation of TMV rods has previously been recorded to occur during the isolation and purification of wild-type TMV (Symington et al., 1962; Steere, 1963). Attempts to disrupt this by sonication proved to be ineffectual when applied to the TMV nanorods described here.

### Distinct Length Classes of Nanorods Can Be Separated by Sucrose Gradient Ultracentrifugation

**FIGURE 3:**
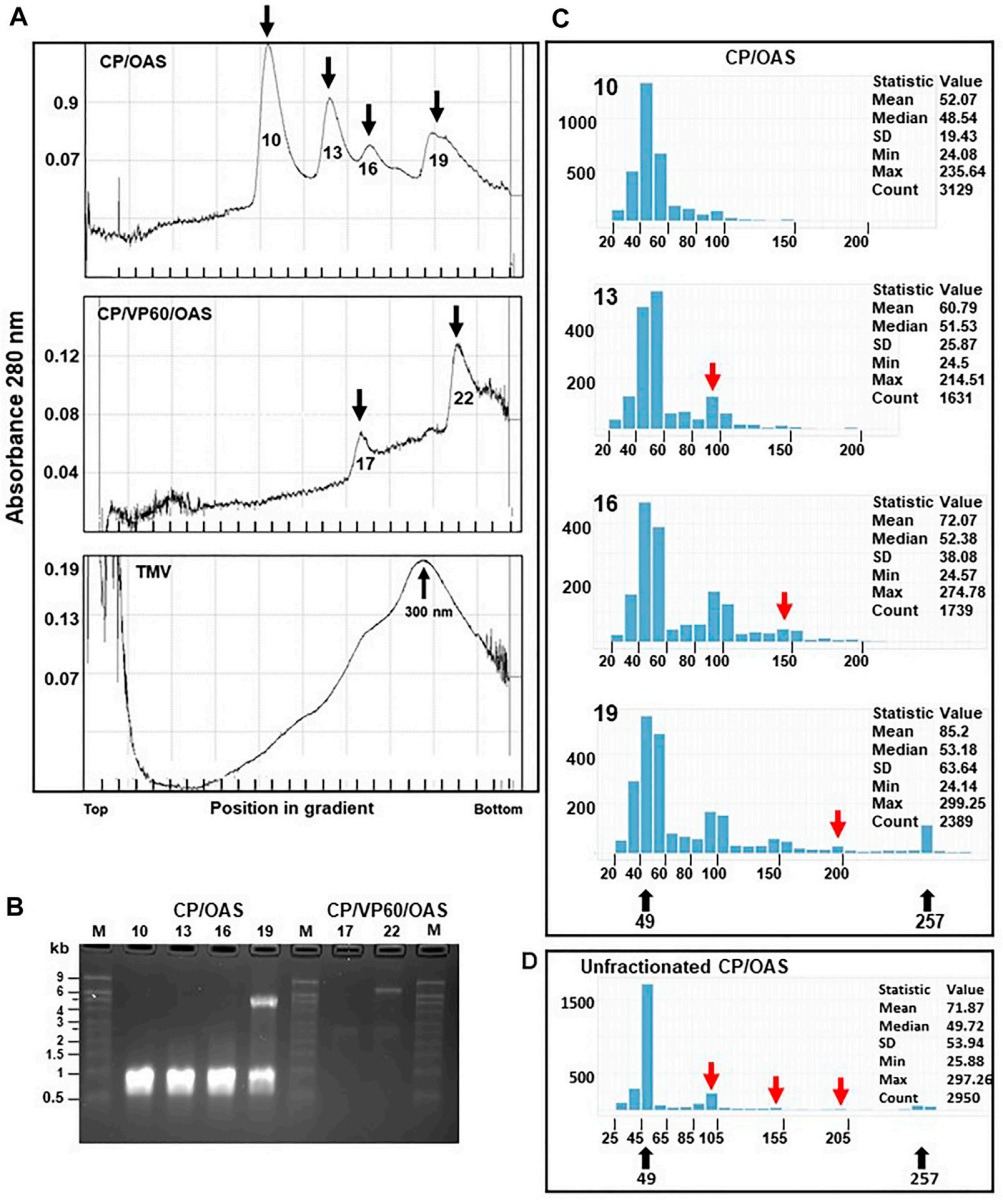
Distinctive size classes of nanorods can be separated by sucrose-density gradient ultracentrifugation centrifugation. **(A)** CP/OAS nanorods, upper panel, CP/VP60/OAS nanorods, middle panel and TMV lower panel separated on a 15–30% sucrose density gradient and fractionated. *Y* axes, absorbance (280 nm) measured during fractionation. Fractions, 10, 13, 16 and 19 (*X* axis) corresponding to measurement peaks are marked by arrows, upper panel, and similarly fractions 17 and 22, middle panel. Lower panel, TMV with the virus peak migration indicated at 300 nm. **(B)** Ethidium bromide-stained denaturing agarose gel of RNA extracted from fractions indicated in **(A)**. M, RNA size ladder. **(C)** Histograms, output of the Nanorod UI interface, showing the length distribution of nanorods from computer-automated measurements of TEM images for CP/OAS fractions 10, 13, 16 and 19. The nanorod expected lengths 49 and 257 nm are indicated below. *Y* axes, number of nanorods in each size class; *X* axes, length distributions in 10 nm increments. The expected length of nanorods derived by end-to-end formation of 49 nm nanorods is shown, red arrows. The descriptive measurement statistics (including mean, median, standard deviation; minimum and maximum length and total count) is shown for each histogram. **(D)** Histogram showing the length distribution of particle lengths from computer-automated measurements of TEM images for CP/OAS unfractionated nanorods.

To develop methods for nanorod characterization, purified nanorods were subjected to ultracentrifugation through 15–30% (w/v) sucrose gradients followed by fractionation. Given that RNA analysis (Figure 2C) showed that only two discrete lengths of RNA were encapsidated, each sample was expected to be resolved into two major peaks. While this appeared to be the case with CP/VP60/OAS nanorods (Figure 3A, middle panel), the gradient profile of CP/OAS nanorods showed four distinct peaks (Figure 3A upper panel). As a control, a preparation of TMV virions that had been stored under cold room conditions for over 40 years, was analysed and gave a major peak that migrated four fifths the way down the sucrose gradient (Figure 3A lower panel). This migration corresponded to full-length 300 nm virus rods, though some degradation in the preparation, indicated by the presence of more slowly migrating material, could be seen. To determine the origin of each of the fractions seen in Figure 3A, RNA was extracted from the nanorods in each fraction and examined by electrophoresis on denaturing agarose gels (Figure 3B). For CP/OAS nanorods, the first three fractions from the top of the gradient (10, 13 and 16) all contained RNA of the same length (approximately 1.0 kb), corresponding to the smaller RNA in unfractionated preparations (Figure 2C). This is consistent with the first three fractions containing monomeric, dimeric and trimeric versions of the 49 nm nanorods, with calculated lengths of 49, 98 and 147 nm, respectively, with the more rapidly sedimenting forms being the products of end-to-end aggregation. Nanorods in Fraction 19 contained a mixture of RNAs of 1 and 5.5 kb. This pattern could result from this fraction containing a mixture of the 257 nm and multimers of the 49 nm nanorods or, indeed, end-to-end aggregates of the two sizes of nanorods. In the case of CP/VP60/OAS, the nanorods in fraction 17 contained RNA of approximately 2.5 kb, while two RNA molecules of approximately 2.5 and 7.0 kb were found in nanorods from fraction 22. These sizes are consistent with those seen in Figure 2C and suggests that the faster sedimenting peak contains a mixture of different length nanorods, while the slower migrating contains exclusively nanorods with a predicted size of 129 nm.

To examine the morphology of the nanorods in the four fractions from the CP/OAS sucrose gradient, samples were analysed by electron microscopy using negative staining. Measurements of nanorod lengths was achieved by utilising single particle analysis software to perform accurate and reproducible micrographs. This approach greatly increased the number of particles that were analysed and eliminated human errors that can occur with repetitive manual measuring procedures. These measurements were carried out by a computer methodology utilising a custom-written Python-based computer script employing selected TEM grid areas, referred to as “GridSquares”. Note, a GridSquare is the area examined by the computer script, not the area between the physical bars on the copper grid which is much larger. Incorrectly identified nanorods, potentially due to overstaining and the presence of contaminating proteinaceous material were not used and rejected from the nanorod count. In some instances, not all nanorods were identified as shown in Supplementary Figure S1A (blue arrows, left panel), and some “rogue” nanorods, consisting of joined side-by-side entities were sometimes recorded (red arrows). To overcome this anomality, these rogue nanorods, consisting of 276 entries, were removed manually from this measurement count. The resulting histogram generated by the Nanorod UI interface, resulted in a similar class profile to that originally measured (compare the upper and lower panels of Supplementary Figure S1B) so consequently this manual “cleaning” step, open to human errors, was not routinely adopted. Similarly, Supplementary Figure S1C presents CP/OAS nanorod length distributions determined from eight different independent GridSquare locations on a single TEM grid. The measurement profile and statistical data of any GridSquare is similar to any one of its neighbours, indicating little variation in class lengths over the entire TEM grid surface and hence supports our confidence in the measurements obtained by our computer methodology. Nanorods of length 40–50 nm were the predominant size class in all CP/OAS fractions, upper panel, Figure 3C. This corresponds well to the predicted length of 49 nm resulting from nanorod formation on the 1.0 kb subgenomic RNA. While this was anticipated with Fraction 10, the occurrence of large amounts of this size class in the faster sedimenting fractions 13, 16 and 19 was more surprising. It suggests that the longer nanorods resulting from end-to-end aggregation, although surviving the conditions of sucrose gradient ultracentrifugation, are disrupted into their constituent parts when subjected to the harsher conditions of negative staining and electron microscopy. This is supported by the electron microscope analysis of the particle lengths in the CP/OAS unfractionated sample, Figure 3D where the major length class consists of 49 nm rods with far lower amounts of the longer forms than suggested by the sucrose gradient analysis. However, nanorods of 257 nm, resulting from the encapsidation of the 5.5 kb RNA are clearly present in the unfractionated sample.

### Production of Nanorods With Modified Coat Proteins

**FIGURE 4:**
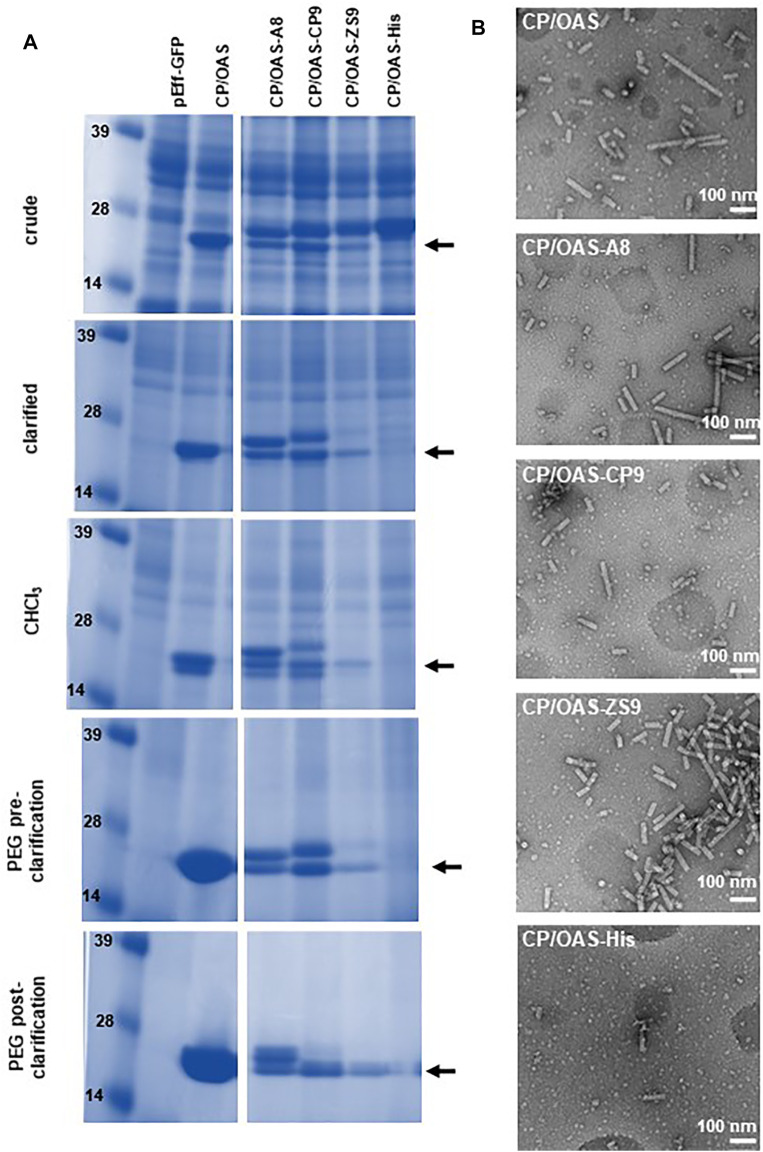
pEff expression of coat-protein variants. **(A)** Instant Blue stained NuPAGE-MOPS gel electrophoresis of wild-type and variant CPs at different stages of the nanorod purification process as indicated. Further details can be found in Supplementary Figure S2. The position of wild-type CP is indicated by an arrow. All variant CPs resolve above this band. **(B)** Transmission electron microscopy images of 118,00 x *g* pellets. Scale bar = 100 nm. Nanorod samples are indicated in each panel.

Previously, it had been shown that the non-replicating pEAQ-*HT* system could be used to generate nanorods with coat proteins displaying a six amino acid, cobalt-platinum binding peptide, -CNAGDHANC, at their C-termini (Saunders and Lomonossoff, 2017). Although functional at metal binding, the yield of nanorods was low and it was not possible to produce significant yields of nanorods containing other peptides using this approach. To examine whether the pEff -based approach described above could address this issue, the sequence of TMV CP was modified to display C-terminal peptides with different metal binding properties (Figure 1C). These modified CP sequences were substituted into pEff-CP/OAS in place of the wild-type CP sequence and the modified constructs infiltrated into *N. benthamiana* leaves. The accumulation of the modified subunits compared to wild-type CP was monitored at each stage of the nanorod purification protocol (Figure 4A). Each variant CP appeared to accumulate to a similar level in crude extracts, though some material running at the size of wild-type CP could also be seen (arrowed), presumably because of loss of the displayed peptide by proteolysis. As purification proceeded, the proportion of apparent wild-type CP in each preparation increases while the overall yield of CP decreases (Figure 4A). The ability of the modified CPs to be incorporated into nanorods was assessed by transmission electron microscopy of the final step of the purification. In each case, nanorods with expected size of approximately 50 nm could be observed (Figure 4B). However, the yield of purified nanorods, as measured by the BCA assay, varied considerably depending on the peptide expressed (Table 1). The yield of nanorod CP/OAS-A8 and, to a lesser degree, CP/OAS-CP9 was higher than the yield of CP/VP60/OAS nanorods and was sufficient for subsequent downstream applications. Nevertheless, it is clear that even in these cases, only a proportion of the subunits retain the C-terminal modification. It is likely that the insertions at the C-terminus alter the surface properties of the particles, suggesting that modification-specific purification protocols may need to be developed.

**TABLE 1:** Yield of nanorods.

Nanorod	Yield
	Expressed as mg/g Fresh Weight Leaf Material
CP/OAS	1.09
CP/VP60/OAS	0.04
CP/OAS-A8	0.38
CP/OAS-CP9	0.22
CP/OAS-ZS9	0.05
CP/OAS-His	0.02

### Gold Nanoparticles Bind at the Ends of CP/OAS-A8 Nanorods

**FIGURE 5:**
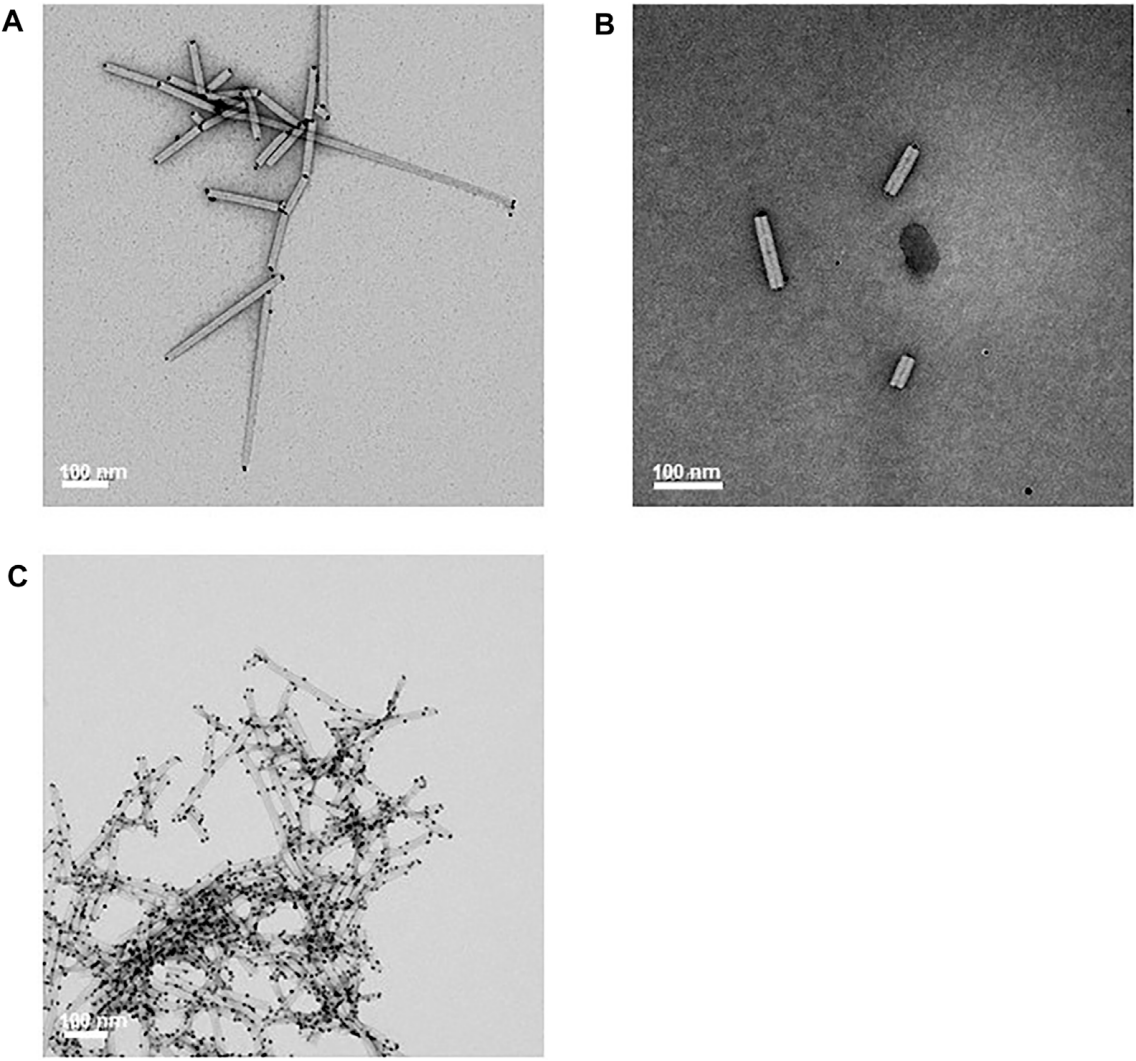
Stained transmission electron microscopy images. **(A)** and **(B)** shows selective gold binding to the ends of CP/OAS-A8 nanorods. **(C)** The effect of an excess (20-fold) of colloidal gold on the binding to the nanorod outer surface in 5 mM citric acid. Scale bars 100 nm as shown.

The ability of unfractionated CP/OAS-A8 nanorods to bind citrate-stabilized colloidal gold particles 5 nm in diameter was examined since the A8 peptide–VSGSSPDS had previously shown to promote the binding of colloidal gold when expressed on the surface of bacteriophage (Huang et al., 2005; Peele et al., 2005). Colloidal gold surface binding was evident only at the CP/OAS-A8 nanorod ends, and most of the nanorod surface was free from excessive gold nanoparticles (Figures 5A,B). The binding of gold nanoparticles to one, both or neither end of the nanorods was measured by transmission electron microscopy of an area of a few hundred nm^2^ of each grid. For CP/OAS-A8, the vast majority (>95%) of nanorods showed gold binding at both ends and did not show a single nanorod without gold binding (Supplementary Figure S3). By contrast, CP/OAS nanorods containing wild-type (wt) CP, and wt TMV particles showed reduced colloidal gold binding, that occurred predominantly at only one end of the rod, under similar reaction conditions (Supplementary Figure S4). Exposure of CP/OAS-A8 nanorods to a twenty-fold excess of colloidal gold nanoparticles in 5 mM citric acid resulted in non-specific binding of the gold nanoparticles on the nanorod surface (Figure 5C).

## Discussion

The results presented here confirm and extend our previous work that suggested that the pEff vector system (Mardanova et al., 2017) would be suitable for the production of TMV-based nanorods of defined length (Thuenemann et al., 2021). A major advantage of the pEff approach is the yields that can be obtained which we estimate to be approximately 2,500 higher than that previously reported for the equivalent nanorods produced using a non-replicating system (Saunders and Lomonossoff, 2017). As previously reported, the yield of nanorods is inversely proportional to their length. In the case of pEff-produced material this may reflect the fact that increasing the length of the PVX replicon is known to decrease its replication competence (Avesani et al., 2007), leading to there being less RNA available to act as a scaffold for particle assembly.

Apart from the large gain in yield, a major difference between the results presented here and those of Saunders and Lomonossoff (2017), is that the use of the pEff vector results in the encapsidation of two sizes of RNAs, derived from the genomic and subgenomic RNAs. For some applications the use of a mixed population may not be problematic; however, for others it may be necessary to use nanorods of a single length. Sucrose density gradient centrifugation, coupled with computer-assisted length measurements, showed that it is possible to separate nanorods into different size classes. However, the gradient profiles were more complex than anticipated due to a tendency of the nanorods to aggregate end-to-end. This tendency needs to be taken into account when fractionating nanorod populations. However, the aggregates can clearly be disrupted under certain conditions, such as those that occur during negative staining. Thus, it may be possible to devise conditions that either prevent the aggregates for forming or enable their subsequent disruption.

The incorporation of genetically modified CP subunits into TMV particles has been extensively investigated, most often using infectious TMV (Petukova et al., 2014; Love et al., 2015; Lomonossoff and Wege, 2018), We have shown that it is possible to incorporate subunits with metal-binding peptides as C-terminal extensions into nanorods produced via pEff-based expression. As has been found previously, the display of heterologous sequences can make purification problematic, and the inserted peptide tends to be lost through proteolysis. Colloidal gold binding was evident at both ends of the CP/OAS-A8 nanorods where the A8 amino acid addition would be expected to be more prominently displayed than on the nanorod cylinder surface. The lower level of gold binding observed with CP/OAS and wt TMV may result from interaction with RNA exposed at the ends of the rods. Gold nanoparticles are known to conjugate to free RNA (Huixiang and Lewis, 2005; Giljohann et al., 2009) and RNA exposed at the end of TMV particles has been shown to bind colloidal gold (Balci et al., 2007).

Our colloidal gold nanoparticles remained bound to CP/OAS-A8 nanorods even after several months’ storage at 4°C, without any loss of the particle architecture. Gold nanoparticles, nanorods and nanowires are an exceptional inorganic material ideal for research encompassing bio-nano engineering and medical applications. The ability to produce large amounts of nanorods using the methods described in this manuscript opens up opportunities for their use in bionanotechnology. For example, the ability to control the length of the nanorods increases their potential as templated drug carriers, where shape and size are a factor for efficient drug delivery. The selective length and metallization of the nanorods reported here could increase their deployment as nanocomposite materials for various applications in nanoelectronics (Venkataraman and Hefferon, 2021).

## Data Availability

The original contributions presented in the study are included in the article/Supplementary Files, further inquiries can be directed to the corresponding author.

## Supplementary Material

Supplementary Figure S1 Measurement of nanorods. **(A)** Transmission electron microscopy image of CP/OAS nanorods. Left panel UA-stained image. Clustered nanorods incorrectly acknowledge to be nanorods, red arrows and nanorods not recognised, blue arrows. Right panel numbered nanorods identified by computational analysis. **(B)** Distribution of all measured nanorods, upper panel. Lower panel measured distribution corrected with removal of incorrectly identified or rogue nanorods. **(C)** Variation of measured nanorod lengths between eight individual GridSquares of CP/OAS, gradient fraction 13, Figure 3A. Measurements were made of particles isolated from pEff-CP/OAS infiltrated leaves. Y axes, measurement length in nm; X axes, length distribution in 5 nm increments. The descriptive statistics for each GridSquare is presented.

Supplementary Figure S2 NuPAGE-MOPS gel electrophoresis of the various nanorods at the different stages of isolation.

Supplementary Figure S3 Colloidal gold nanoparticles binding on CP/OAS-A8 nanorods was measured by transmission electron microscope. Scale bars as shown.

Supplementary Figure S4 Control experiments for colloidal gold nanoparticles binding on **(A)** CP/OAS and **(B)** wt TMV was measured by transmission electron microscope images respectively. Scale bars as shown.
