# Supplementary figures and images for: The Use of a Replicating Virus Vector For in Planta Generation of Tobacco Mosaic Virus Nanorods Suitable For Metallization
Source: Front Bioeng Biotechnol. 2022 Apr 26;10:877361. doi: 10.3389/fbioe.2022.877361 (PMC9086362; doi:10.3389/fbioe.2022.877361)

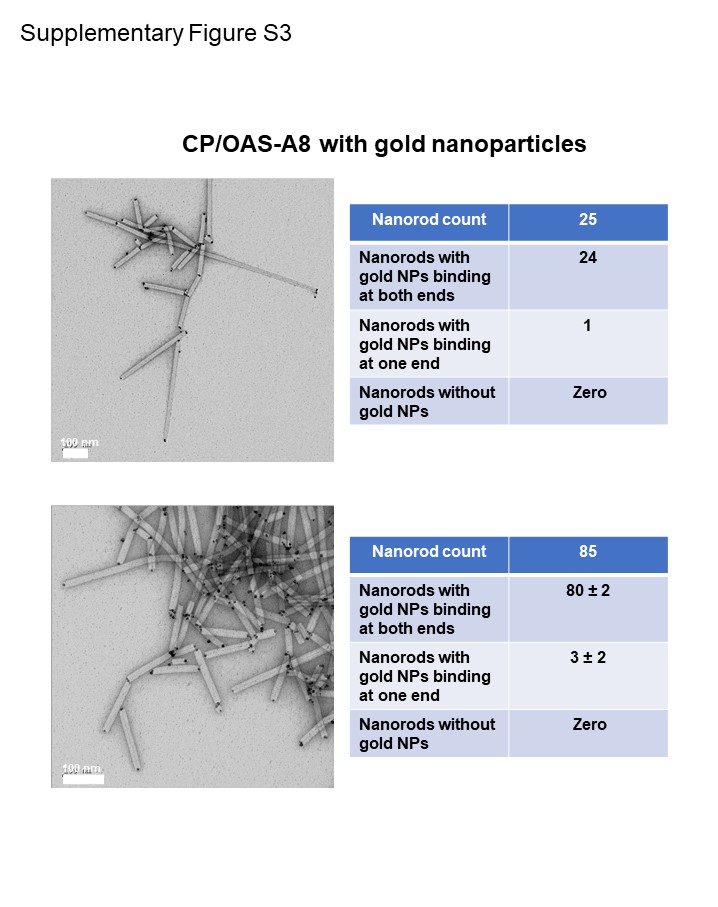

Supplement: Supplementary file 1 [file Image3.JPEG]

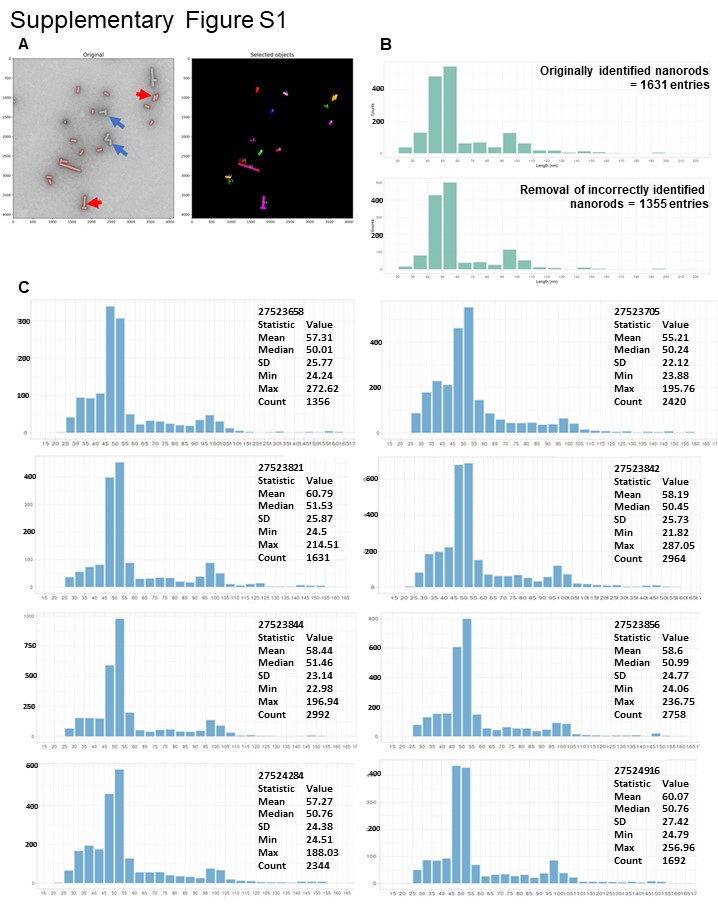

Supplement: Supplementary file 2 [file Image1.JPEG]

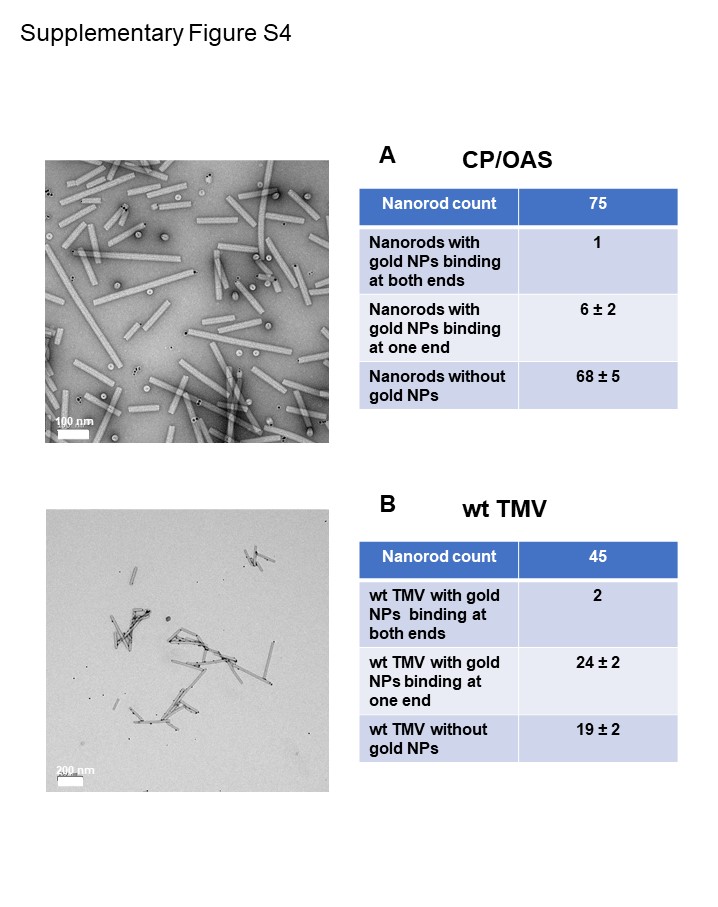

Supplement: Supplementary file 3 [file Image4.JPEG]

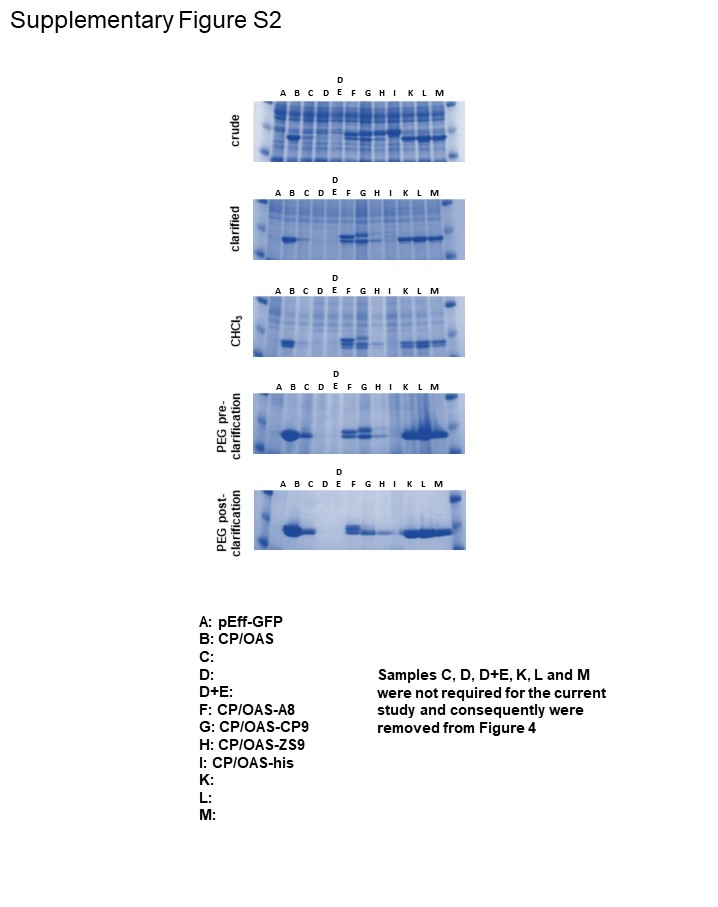

Supplement: Supplementary file 4 [file Image2.JPEG]
